# Urolithins at the Crossroads of Gut Inflammation and Cancer—A Narrative Review

**DOI:** 10.3390/nu18142253

**Published:** 2026-07-10

**Authors:** Anna Duda-Madej, Szymon Viscardi, Jakub Łabaz, Hanna Bazan, Marta Szandruk-Bender

**Affiliations:** 1 Department of Microbiology, Faculty of Medicine, Wroclaw Medical University, Chałubińskiego 4, 50-368 Wrocław, Poland; 2 Department of Pharmacology, Faculty of Medicine, Wroclaw Medical University, Mikulicza-Radeckiego 2, 50-345 Wrocław, Poland; szymon.viscardi@student.umw.edu.pl (S.V.); marta.szandruk-bender@umw.edu.pl (M.S.-B.); 3 Faculty of Medicine, Wroclaw Medical University, Ludwika Pasteura 1, 50-367 Wrocław, Poland; jakub.labaz@student.umw.edu.pl (J.Ł.); hanna.bazan@student.umw.edu.pl (H.B.)

**Keywords:** colorectal cancer, diet–gut microbiota–immune system–cancer axis, ellagic acid, ellagitannins, gut microbiota, inflammatory bowel disease, postbiotics, urolithins

## Abstract

Chronic inflammation and the associated dysbiosis of the gut microbiota are increasingly recognized as key factors contributing to the development of inflammatory bowel disease (IBD) and colorectal cancer (CRC). The diet–gut microbiota–immune system–cancer axis is considered a key regulator of these processes. Among the bioactive compounds found in the diet, ellagitannins have garnered significant scientific interest due to their conversion by the gut microbiota into biologically active metabolites known as urolithins. Urolithin A (UroA), one of the best characterized compounds, exhibits broad anti-inflammatory, antioxidant, immunomodulatory, and anticancer properties. It modulates signaling pathways associated with inflammation, oxidative stress, mitochondrial dysfunction, and cell proliferation. Furthermore, UroA has been shown to improve intestinal barrier integrity, regulate immune cell activity, and induce mitophagy, thereby contributing to the restoration of mitochondrial and cellular homeostasis. A growing body of evidence also suggests that UroA may inhibit cancer cell proliferation, induce apoptosis, and disrupt the molecular pathways involved in colorectal carcinogenesis. This review summarizes the current state of knowledge regarding the biosynthesis and bioavailability of UroA, its molecular mechanisms of action in IBD, and its potential role in the prevention and treatment of CRC. Furthermore, the limitations of UroA-based therapies and future research directions are discussed. Although further, well-designed clinical trials are necessary, current findings suggest that UroA may represent a promising microflora-targeted therapeutic strategy in chronic inflammatory and CRC diseases.

## 1. Introduction

In recent years, increasing attention has been paid to the relationships between the gut microbiota and chronic inflammation or the development of cancer. The focus of current research is on the diet–gut microbiota–immune system–cancer axis, whose function can be modulated by both environmental and dietary factors [1,2,3]. By influencing the functioning of the gut microbiome, diet therefore plays a key role in maintaining the health of the entire body [4]. Unhealthy eating habits may lead to disturbances in the composition and function of the gut microbiota, weakening of intestinal barrier integrity, and, consequently, prolonged activation of the immune response. Chronic inflammation is currently recognized as one of the key mechanisms involved in the initiation and progression of cancers, particularly within the gastrointestinal tract [5,6]. Furthermore, the associated excessive immune response promotes disturbances in tissue homeostasis [7,8], increased oxidative stress [9], DNA damage [10,11], and the dysregulation of pathways controlling cell proliferation and death [12,13]. This correlation is particularly dangerous in the progression of inflammatory bowel disease (IBD), in which chronic inflammation increases the risk of developing colorectal cancer (CRC) [14,15,16]. Therefore, the growing incidence of IBD and gastrointestinal cancers has intensified interest in bioactive compounds capable of simultaneously modulating inflammatory and carcinogenic processes, potentially contributing to the prevention and supportive treatment of chronic diseases. Particular attention is directed towards postbiotics, i.e., metabolites of microbial origin [17,18].

An increasing number of studies indicate that naturally derived bioactive compounds not only influence the composition of the gut microbiota [19]. They may also modulate the activity of molecular pathways associated with the immune response [20,21] and oxidative stress [22,23,24], as well as influencing the integrity of the intestinal barrier [25] and cell proliferation [24,26]. Among these compounds, polyphenols are of particular importance, as their regular consumption has been associated with a reduced risk of developing chronic inflammatory diseases and cancer [27,28].

Among bioactive compounds, ellagitannins have attracted considerable scientific interest. These high-molecular-weight polyphenols are present in pomegranates, raspberries, strawberries, blackberries, and walnuts [29]. In addition to their antibacterial, antiviral, anti-atherosclerotic, anticancer, and antidiabetic properties [30,31,32,33,34], they also exhibit very strong antioxidant and anti-inflammatory activity [35,36]. However, due to their limited bioavailability in the human body, key biological significance is attributed to the products of their metabolism formed with the participation of the gut microbiota. In the gastrointestinal tract, ellagitannins are hydrolyzed to ellagic acid (EA), which is subsequently converted by commensal gut bacteria into a group of metabolites known as urolithins [37]. The ability to produce urolithins differs between individuals and depends on the qualitative and quantitative composition of the gut microbiota, which has led to the identification of so-called urolithin metabotypes [38]. The involvement of the gut microbiota in the formation of these postbiotics highlights their importance in maintaining the body’s homeostasis and regulating disease-related processes. Current research indicates that urolithins exhibit a range of potential health benefits, particularly in the context of diseases associated with chronic inflammation [34,39]. Although this review focuses primarily on urolithin A (UroA), evidence on other urolithins, EA, ellagitannins, or pomegranate-derived preparations is discussed only when it provides mechanistic or metabolic context. Such findings are explicitly identified throughout the manuscript and should not be interpreted as direct evidence of purified UroA. These compounds have been shown to possess anti-inflammatory, antioxidant, immunomodulatory and intestinal barrier-protective properties [40,41,42,43]. Furthermore, they have been implicated in the regulation of signaling pathways associated with the inflammatory response and in the reduction in pro-inflammatory cytokine production. Moreover, their beneficial effect on mitochondrial function and processes related to cellular homeostasis has also been observed, which may indicate their role in preventing tissue damage and the development of chronic diseases [44].

**Figure 1:**
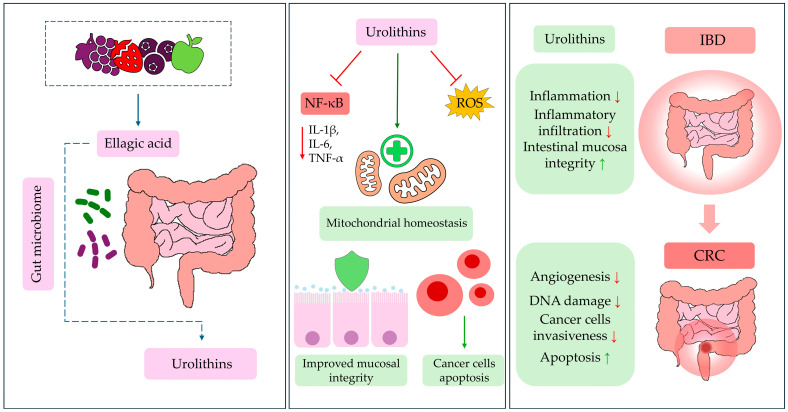
A scheme of the influence of urolithins associated with the diet–gut microbiota axis on chronic inflammation and carcinogenesis in the gastrointestinal tract. The proposed anti-inflammatory mechanisms are based on the studies summarized in Section 5.1, Section 5.2, Section 5.3, Section 5.4, Section 5.5 and Section 5.6 [43,48,49,50,51,52,53,54,55,56,57]. The proposed anticancer mechanisms are based on the studies discussed in Section 6.2, Section 6.3, Section 6.4, Section 6.5 and Section 6.6 [46,56,58,59,60,61,62,63]. Ellagitannins derived from fruits and nuts are biotransformed by the gut microbiota into ellagic acid and subsequently converted into urolithin metabolotypes. Urolithins exhibit anti-inflammatory and antioxidant effects by inhibiting NF-κB (nuclear factor κB) signaling, reducing the production of pro-inflammatory cytokines (IL-1β, IL-6, TNF-α), decreasing oxidative stress (ROS), and improving epithelial barrier integrity and mitochondrial function. Additionally, they induce apoptosis and inhibit the proliferation of cancer cells. As a result, these mechanisms contribute to reducing inflammation and improving intestinal barrier function in the course of inflammatory bowel disease (IBD). At the same time, by reducing DNA damage, limiting angiogenesis, and inhibiting tumor invasion, they inhibit the initiation and progression of colorectal cancer (CRC). Symbols: ↑—indicates increase; ↓—indicates decrease; ┴—indicates inhibition.

Interestingly, a growing number of studies indicate that urolithins can play a noticeable role in modulating carcinogenic processes [45,46]. Experimental studies have demonstrated their ability to inhibit cell proliferation, induce apoptosis, and modulate the molecular pathways involved in cancer development and progression [47]. Their biological activity appears to be closely associated with gut microbiota homeostasis, further highlighting the importance of microbial metabolism in maintaining human health. Figure 1 illustrates the modulation of the diet–gut microbiota–immune system–cancer axis by urolithins and their role in restoring intestinal and immune homeostasis, reducing chronic inflammation, and potentially protecting against colorectal carcinogenesis.

A recent increase in the number of studies suggests that urolithins may represent a promising class of compounds capable of modulating processes associated with chronic inflammation and carcinogenesis within the gastrointestinal tract. This article provides an overview of the current understanding of the molecular mechanisms of action of these ellagitannin metabolites, which are produced with the involvement of the gut microbiota. Particular attention is paid to their impact on mechanisms related to the inflammation–carcinogenesis axis, and especially to their role in modulating biological pathways relevant to the pathogenesis of IBD and CRC.

## 2. Materials and Methods

**Figure 2:**
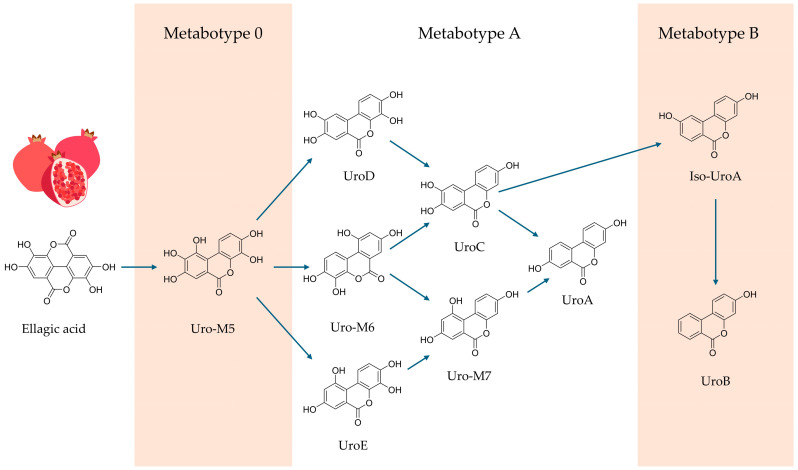
A summary of ellagic acid (EA) biotransformation towards selected urolithins (Uro).

**Figure 3:**
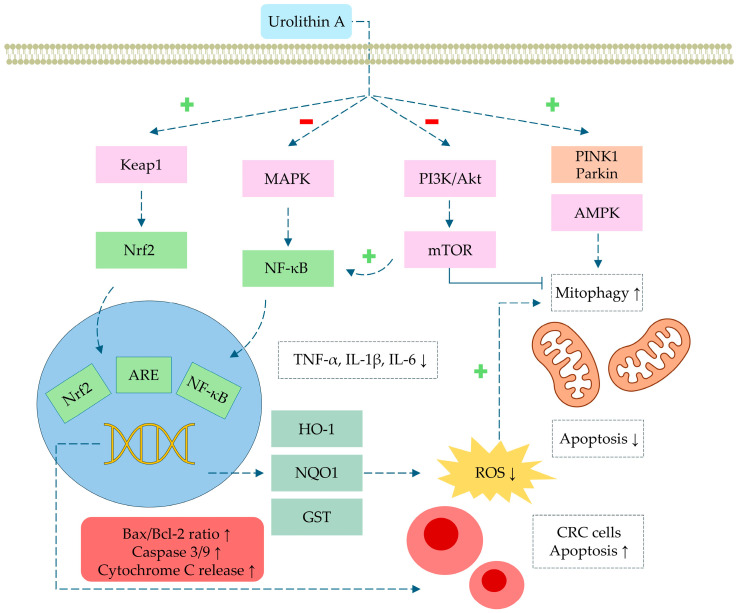
The context-dependent molecular pathways regulated by UroA. In normal and inflamed tissues, UroA predominantly activates mitophagy, antioxidant responses, and cytoprotective signaling while reducing apoptosis. In contrast, in colorectal cancer cells, UroA promotes apoptosis through activation of Bax/Bcl-2-, caspase-, and cytochrome c-dependent pathways, contributing to tumor suppression. Abbreviations: AMPK—5′-AMP-activated protein kinase, ARE—antioxidant response elements, GST—glutathione-S-transferase, HO-1—heme oxygenase 1, IL—interleukin, Keap1—Kelch-like ECH-associated protein 1, MAPK—mitogen-activated protein kinase, mTOR—mammalian target of rapamycin, NF-κB—nuclear factor κB, NQO1—NAD(P)H dehydrogenase, Nrf2—nuclear factor erythroid 2-related factor 2, Parkin—E3 ubiquitin ligase, PINK1—PTEN-induced kinase 1, PI3K/Akt—phosphoinositide 3-kinases/protein kinase B, ROS—reactive oxygen species, and TNF—tumor necrosis factor. Symbols: green plus—upregulation/activation, red minus—downregulation/inhibition, ↑—indicates increase, ↓—indicates decrease, and ┴—indicates inhibition.

**Figure 4:**
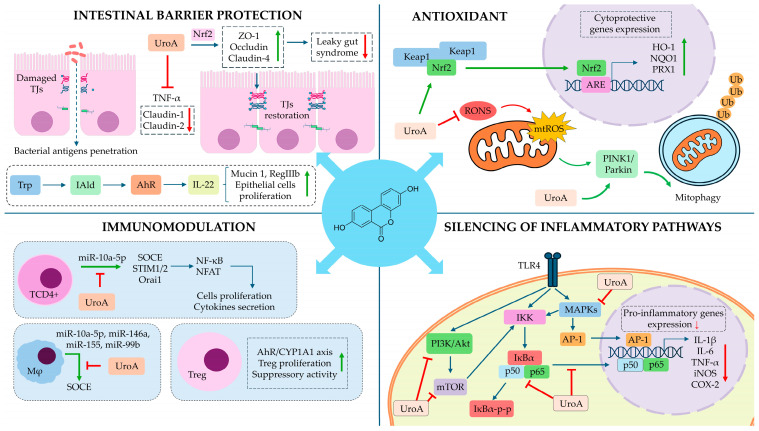
The spectrum of the molecular effects of urolithin A in the alleviation of IBD. Abbreviations: AhR—aryl hydrocarbon receptor, Akt—protein kinase B, AP-1—activator protein 1, ARE—antioxidant response element, COX-2—cyclooxygenase-2, CYP1A1—cytochrome P450 family 1 subfamily A polypeptide 1, HO-1—heme oxygenase-1, IAld—indole-3-aldehyde, IKK—IκB kinase, IL- interleukin, iNOS—inducible nitric oxide synthase, IκBα—nuclear factor of kappa light polypeptide gene enhancer in B cells inhibitor alpha, IκBα-p-p—phosphorylated IκBα, Keap 1—Kelch-like ECH-associated protein 1, MAPKs—mitogen-activated protein kinases, miR—microRNA, mTOR—mammalian target of rapamycin, mtROS—mitochondrial reactive oxygen species, Mϕ—macrophage, NFAT—nuclear factor of activated T cells, NF-κB (IκBα/p50/p65)—nuclear factor kappa-light-chain-enhancer of activated B cells, NQO1—NAD(P)H Quinone Dehydrogenase 1, Nrf2—nuclear factor erythroid 2-related factor 2, PI3K—phosphoinositide 3-kinase, PINK1—phosphatase and tensin homolog-induced kinase 1, PRX1—paired related homeobox 1, RegIIIb—regenerating islet-derived protein 3 beta, RONS—reactive oxygen and nitrogen species, T CD4+—T helper cells, TJs—tight junctions, TLR4—toll-like receptor 4, TNF-α—tumor necrosis factor alpha, Treg—regulatory T cell, Trp—tryptophan, Ub—ubiquitin, UroA—urolithin A, and ZO-1—zonula occludens-1. Symbols: ↑—indicates increase; ↓—indicates decrease; ┴—indicates inhibition.

**Figure 5:**
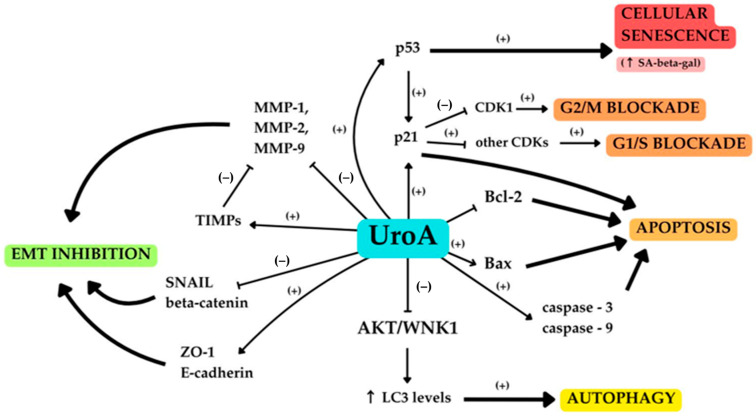
A summary of UroA anticancer activity in CRC. Abbreviations: AKT—protein kinase B; Bax—Bcl-2-associated X protein; Bcl-2—B cell lymphoma 2 protein; CDK1/CDKs—cyclin-dependent kinase 1/cyclin-dependent kinases; E-cadherin—epithelial cadherin; EMT—epithelial–mesenchymal transition; G1/S—gap 1 phase/synthesis phase; G2/M—gap 2 phase/mitosis phase; LC3—microtubule-associated protein 1A/1B-light chain 3; MMP-1, MMP-2, MMP-9—matrix metalloproteinases 1, 2, 9; p21—protein 21/cyclin-dependent kinase inhibitor 1; p53—tumor protein p53; SA-beta-gal—senescence-associated beta-galactosidase; SNAIL—zinc finger protein SNAI1; TIMPs—tissue inhibitors of metalloproteinases; UroA—urolithin A; WNK1—With No Lysine kinase 1; ZO-1—zonula occludens-1 protein. Symbols: ↑—indicates increase; ↓—indicates decrease; ┴—indicates inhibition.

A literature search was conducted using PubMed, Scopus, and Web of Science databases. Keywords included ‘urolithins’, ‘urolithin A’, ‘inflammatory bowel disease’, and ‘colorectal cancer’. Only English-language articles were included. Studies were selected based on relevance, recency, and methodological quality. A total of 164 articles were included in the final analysis. Figure 1 presents the origin of urolithins and their main molecular effects associated with the alleviation of IBD and the inhibition of tumorigenesis. Figure 2 depicts the biotransformation of ellagic acid via the gut microbiota and three main metabotypes of the mentioned compound. Figure 3 presents the context-dependent molecular pathways regulated by UroA. Figure 4 depicts the molecular effects of urolithin A activity leading to IBD alleviation. Figure 5 presents an in-depth analysis of urolithin A interference with colorectal cancer cells’ molecular pathways. Table 1 presents the results of studies on the effect of UroA on IBD, while Table 2 presents the results of studies on the effect of UroA on CRC.

## 3. Biosynthesis and Bioavailability of UroA

UroA is a gut microbiota-derived postbiotic that has a number of beneficial interactions with the mucosa and the immune system and, following systemic absorption, can exert various metabolic effects at the level of specific organs, e.g., the pancreas, central nervous system, and skeletal muscles [64,65,66,67]. UroA is not directly present in foods but is generated through the microbial transformation of ellagitannins, particularly EA. It is found in various fruits, including pomegranates (the most important source), strawberries, raspberries, and grapes, as well as walnuts and almonds [68,69,70].

A major limitation of EA is its low bioavailability following oral administration, resulting in minimal systemic absorption and its subsequent transit to the colon, where it undergoes microbial metabolism. This process is primarily mediated by specialized ellagitannin-metabolizing bacteria, including *Gordonibacter* spp., *Ellagibacter* spp., and selected *Enterocloster* spp., which catalyze the sequential dehydroxylation and conversion of EA into intermediate urolithins and ultimately UroA. In contrast, other gut bacteria, including *Lactiplantibacillus plantarum*, *Enterococcus faecium*, *Bifidobacterium pseudocatenulatum*, and *Streptococcus thermophilus*, are not considered primary urolithin producers. Instead, they may indirectly support urolithin production by modulating gut microbial ecology, improving intestinal environmental conditions, or promoting the growth and metabolic activity of urolithin-producing bacteria [55,56,57,58,59,60]. The formation of specific EA derivatives is variable individually, with the limiting factor being mainly the qualitative composition of the gut microbiota. Those differences in constitution have led to three main metabotypes of EA being identified. Metabotype A allows for the formation of mainly UroA and its conjugates, metabotype B is characterized by the synthesis of UroB, UroA, and iso-urolithin A (iso-UroA), and metabotype 0 is not capable of forming any urolithins (Figure 2) [71,72,73]. From a clinical perspective, individuals classified as metabotype 0 are unlikely to derive the same benefits from ellagitannin-rich foods because they are unable to efficiently convert dietary precursors into bioactive urolithins. This considerable interindividual variability may partly explain the various biological responses reported in nutritional intervention studies. Moreover, the composition of gut microbiota is influenced by numerous secondary factors: diet, inflammation, and age. Dysbiosis typical of diseases such as IBD or cancer (e.g., CRC) may therefore be a factor limiting the formation of UroA [72,74,75]. This can once again lead up to unforeseen results. Unfortunately, there are currently no studies researching exactly how effective oral supplementation of UroA is compared to natural production. Following absorption, UroA undergoes phase II metabolism in both enterocytes and the liver—known as glucuronidation and sulfation. Conjugated metabolites may then be excreted in urine or feces as a result of enterohepatic circulation [76,77].

Pharmacokinetic studies indicate that all forms (conjugated and free) reach peak plasma concentrations (T_max_) at approximately 6 h; free UroA and its glucuronide are characterized by T_1/2_ = 17–22 h and UroA-sulfate T_1/2_ = 25–58 h, while complete elimination from plasma lasts 72–96 h from the last dose of the preparation [78]. In plasma, the dominant forms are glucuronide, sulfate and free UroA, which have been detected in various tissues such as the liver, colon, lungs or kidneys, with evidence suggesting that the aglycone form may cross the blood–brain barrier [79,80,81]. Importantly, the conjugated form is characterized by lower biological activity, but the phenomenon of local deconjugation, e.g., in inflamed tissues with higher β-glucoronidase activity, increases the biological efficiency of UroA [82,83]. Although most mechanistic studies use free UroA, the predominant circulating forms in humans are glucuronide and sulfate conjugates. Increased β-glucuronidase activity in inflamed tissues may locally regenerate free UroA, allowing biologically active concentrations to be achieved directly at sites of inflammation. Therefore, plasma concentrations of conjugated metabolites may not accurately reflect the amount of biologically active UroA present within inflamed tissues.

In patients with CRC, supplementation with pomegranate extract resulted in the detection of multiple urolithin derivatives in colon tissue, although their concentrations varied substantially between individuals. Notably, a higher punicalagin-to-EA ratio was associated with reduced UroA production, suggesting that precursor composition may considerably influence microbial metabolism [84]. Because pomegranate extract contains multiple ellagitannins and other polyphenols, these observations cannot be attributed exclusively to UroA and likely reflect combined effects of precursor compounds and their microbial metabolites.

Overall, the bioavailability of UroA is highly dependent on interindividual variability in gut microbiota composition, which significantly limits the predictability of its systemic exposure and biological effects. Moreover, although pharmacokinetic profiles have been relatively well characterized, the translational relevance of circulating conjugated forms versus locally deconjugated aglycone remains insufficiently understood. Importantly, biological activities reported for ellagitannins, EA, pomegranate extracts, or other urolithins cannot be directly extrapolated to purified UroA because these compounds differ in bioavailability, metabolism, and molecular targets. Therefore, mechanistic evidence discussed in subsequent sections is identified according to the specific compound investigated whenever applicable. Direct UroA supplementation may reduce the limitations associated with differences in gut microbiota composition and urolithin metabotypes. Unlike ellagitannins, UroA does not require microbial conversion before absorption. However, more clinical studies are needed to determine whether direct supplementation provides comparable benefits across different metabotypes.

## 4. Selected Biological Activities of Urolithin A

### 4.1. Mitochondrial Autophagy (Mitophagy)

Mitophagy, a selective form of autophagy targeting mitochondria, plays a crucial role in maintaining cellular homeostasis by removing damaged organelles, thereby preventing the accumulation of dysfunctional mitochondria and reducing oxidative stress [85]. Mechanistically, a signaling pathway dependent on PINK1 and Parkin proteins has been described, leading to mitochondrial ubiquitination and activation of autophagy receptors. UroA has been shown to induce mitophagy and regulate this process (removal of defective or redundant organelles) [78,86]. It also activates the Nrf2 (nuclear factor erythroid 2-related factor 2)/ARE (antioxidant response element) pathway, supporting redox balance via the upregulation of antioxidant enzymes such as glutathione S-transferases, which may further contribute to the regulation of mitophagy through the reduction in oxidative stress [87]. In addition, inhibition of the PI3K/Akt signal cascade by UroA also suppresses mTOR signaling, a key negative regulator of autophagy. By activating autophagy and limiting oxidative stress, UroA contributes to the prevention of apoptosis [88]. These effects have been consistently demonstrated across multiple in vivo models [58,89,90,91]. Notably, the majority of these findings are derived from in vitro and animal models, and therefore, their direct translational relevance to human physiology remains to be established.

### 4.2. Antioxidant and Anti-Inflammatory Properties

UroA exhibits pleiotropic anti-inflammatory and antioxidant effects mediated primarily by the modulation of key cellular signaling cascades. It activates the Nrf2/ARE signaling pathway, a key regulator of cellular antioxidant responses. This activation leads to the upregulation of multiple antioxidant enzymes, including HO-1, NQO1, superoxide dismutase, and catalase, thereby enhancing cellular redox balance. As a result, UroA may contribute to the reduction in ROS accumulation and may protect cells against oxidative stress-induced damage [92,93,94]. Studies also indicate the ability of the substance to inhibit the activity of typical inflammatory pathways, NF-κB, MAPK, and PI3K/Akt, resulting in a decrease in the activity of immune cells and reduced secretion of pro-inflammatory cytokines (e.g., TNF-α, IL-6, IL-1β) [48,95]. There was also a decrease in the synthesis of enzymes important in the pathomechanism of inflammation, e.g., COX-2, iNOS, and mPGES-1 [96,97]. Numerous in vivo models demonstrate the beneficial anti-inflammatory and antioxidant profile of UroA [92,93,98,99]. Overall, UroA exhibits consistent antioxidant and anti-inflammatory activity across experimental models. Although pathway dominance appears context-dependent and remains to be fully validated clinically.

### 4.3. Cell Cycle Regulation

UroA presents pleiotropic effects on cell cycle regulation, being able to induce cell cycle arrest at different phases (G1/S, S/G2, G2/M) and to modulate apoptotic pathways. The previously described autophagy-promoting activity of UroA is associated with the inhibition of apoptosis, primarily through suppression of the PI3K/Akt/mTOR signaling pathway. Conversely, UroA may also promote apoptosis via mechanisms involving an increased Bax/Bcl-2 ratio, activation of caspases (including caspase-3 and caspase-9), the release of cytochrome c, and activation of JNK and p38 signaling pathways [46,63,100]. Importantly, these seemingly opposing effects are context-dependent and vary according to cell type and physiological or pathological conditions. Consistent with this dual role, several in vivo studies have demonstrated cytoprotective effects of UroA, including reversal of the Bax/Bcl-2 ratio, the inhibition of caspase cleavage (e.g., caspase-1 and caspase-3), and activation of the p62(Keap1)/Nrf2/ARE pathway, leading to enhanced mitophagy and antioxidant responses, ultimately promoting cell survival [101,102]. These apparently opposing effects suggest that UroA acts as a context-dependent modulator rather than exerting contradictory biological activities. In normal and inflamed tissues, activation of mitophagy and attenuation of oxidative stress predominantly promote cell survival and tissue homeostasis by limiting apoptosis. In contrast, in colorectal cancer cells, UroA activates pro-apoptotic pathways, including increased Bax/Bcl-2 ratio, caspase activation, and cytochrome c release, thereby suppressing tumor cell survival. This distinction should be considered when interpreting the molecular effects summarized in Figure 3. A summary of the molecular effects of UroA is depicted in Figure 3.

### 4.4. Impact on Metabolic Pathways

Numerous studies highlight the multiple interactions of UroA with key metabolic pathways, including carbohydrate, lipid, and protein metabolism, as well as its pleiotropic effects on cellular energy homeostasis. Through its regulatory effects on mitochondrial function, particularly mitophagy and the reduction in ROS, UroA may enhance the efficiency of ATP production, which is especially important in metabolically active tissues such as skeletal muscle [89]. UroA has also been identified as an activator of AMPK, leading to the stimulation of fatty acid β-oxidation and the inhibition of cholesterol synthesis and gluconeogenesis. These effects have been associated with improvements in lipid profiles and enhanced insulin sensitivity [103,104,105]. In addition, UroA has been shown to modulate peroxisome proliferator-activated receptor gamma (PPARγ) signaling, which plays a key role in lipid metabolism. Through this pathway, UroA can inhibit cholesterol accumulation in oxidized LDL-stimulated foam cells, thereby potentially limiting the progression of atherosclerosis [106]. Furthermore, activation of PPARγ is associated with improved glucose uptake in muscle and adipose tissue, the regulation of lipid synthesis, and the promotion of adipocyte differentiation [107,108]. However, it should be emphasized that most of these metabolic effects have been demonstrated in preclinical models, and their significance and clinical relevance in humans require in-depth studies.

## 5. Molecular Mechanisms Underlying Protective Effects of Urolithin A in Inflammatory Bowel Diseases

IBDs, including Crohn’s disease (CD) and ulcerative colitis (UC), are chronic, destructive conditions with a complex etiology in which genetic predisposition, immune deregulation, intestinal microbiota disorders and epithelial barrier damage play important roles [109]. A central element of IBD pathogenesis is the sustained activation of pro-inflammatory pathways, particularly NF-κB-dependent signaling pathways, leading to overproduction of the IL-1 family (mainly IL-1α and IL-1β), IL-6 and TNF-α, with a simultaneous decrease in anti-inflammatory cytokines, e.g., IL-10 and TGF-β [109,110]. These disorders significantly affect the condition of the human body; chronic inflammation leads to inflammatory arthropathies in the spondyloarthropathies spectrum (7–50%) [111], acute toxic megacolon (1–10%) [112] and the development of cancers, both CRC (approx. 3.2%) and extraintestinal cancers (hematological, skin, urinary tract, cervical cancers), for which an additional risk of development is the use of immunosuppressive drugs in IBD therapy (i.e., thiopurines) [14,113]. It is also worth mentioning that, despite observable therapeutic progress, currently used drugs in IBD still do not allow for long-term remission in the majority of patients; the percentage of remission maintained after 12 months of biologic therapy is often only up to 40% [114]. Furthermore, up to 50% of patients treated with anti-TNF therapy experience a secondary loss of response (LOR) [115]; while primary non-response (PNR) or treatment intolerance is not uncommon with 5-ASA, such as mesalazine, they have a relatively good safety profile during therapy [116].

Due to the above-mentioned problems, there is a need to search for new therapeutic strategies that will effectively control chronic inflammation without increasing the risk of adverse effects, including carcinogenesis. In recent years, significant attention has been paid to the role of gut microbiota metabolites as modulators of the inflammatory response. UroA, a product of ellagitannin metabolism by intestinal bacteria, exhibits a broad spectrum of biological activity, including anti-inflammatory, antioxidant, antiglycemic and neuroprotective properties [117]. In murine experimental models of dextran sulfate sodium (DSS)-induced colitis, UroA has been shown to decrease inflammatory infiltration, lower pro-inflammatory cytokine levels, improve intestinal epithelial integrity and reduce gut microbiome dysbiosis, resulting in lowered disease activity with improved colonic histological changes and colonic prolongation [50,51]. More recent preclinical studies have also confirmed that UroA effectively reduces inflammation in both chemically induced intestinal inflammation (DSS colitis) and immune activation-associated inflammation (immune checkpoint inhibitor-induced colitis) models [118].

The complexity of UroA’s action stems from its ability to simultaneously modulate multiple signaling pathways involved in the pathogenesis of IBD, including PI3K/Akt/NF-κB, MAPK, and Nrf2, which allows, at the same time, for the inhibition of inflammatory processes and activation of AhR-dependent cytoprotective mechanisms [52,119]. This multifaceted action of UroA (described in detail below) makes it a potentially promising candidate in IBD therapy, fitting into current therapeutic strategies aimed at restoring immunological homeostasis in the intestine.

### 5.1. Modulation of Inflammation-Related Signaling Pathways

One of the key mechanisms of UroA’s anti-inflammatory effects is its inhibitory action on NF-κB activation, which plays a main role in regulating the inflammatory response in IBD [48]. Activation of NF-κB p65 leads to the transcription of numerous pro-inflammatory genes (including IL-1β, IL-6 and TNF-α) as well as enzymes such as iNOS and COX-2, making it a mediator between inflammation, IBD, and cancer [48,53]. Studies have shown that UroA inhibits NF-κB activation by limiting IκBα phosphorylation and translocation of the p65 subunit to the cell nucleus, which in turn reduces the expression of the above-mentioned pro-inflammatory cytokines [48,97].

In addition to its direct effect on NF-κB, UroA also indirectly inhibits this transcription factor by affecting intracellular kinase signaling pathways that modulate the cellular response to inflammatory stimuli [48,119]. Activation of the TLR4 receptor by lipopolysaccharide (LPS) leads to initiation of the PI3K/Akt/mTOR and MAPK pathways, which participate in NF-κB mobilization and increased expression of pro-inflammatory cytokines [120,121]. Studies have shown that UroA affects the PI3K/Akt/mTOR pathway, inhibiting the phosphorylation of all its three components, which leads to attenuation of macrophage activation and reduced expression of pro-inflammatory mediators such as iNOS, COX-2, TNF-α, and IL-1β. Inhibition of this pathway also promotes the activation of autophagy, which further reduces the inflammatory response and cellular stress [48,120,122]. UroA also inhibits the activation of MAPKs, particularly p38 and JNK, which are responsible for the transcription of pro-inflammatory genes and increased production of cytokines such as TNF-α and IL-6. This mechanism partially occurs independently of NF-κB, as MAPKs can directly activate transcription factors regulating the inflammatory response [48,121,123].

In addition to the PI3K/Akt/NF-κB and MAPK pathways, the Nrf2 pathway, a major regulator of the cellular antioxidant response, plays an important role in IBD pathogenesis, regulating the inflammatory response and intestinal homeostasis [124]. Under physiological conditions, Nrf2 remains bound to the Keap1 protein in the cytoplasm and is subject to proteasomal degradation [125]. In response to oxidative stress or inflammatory stimuli, Nrf2 is activated and translocated to the cell nucleus, where it binds to AREs (antioxidant response elements)—a cis-acting enhancer sequence found within the promoter region of numerous cytoprotective antioxidant and phase II enzyme genes involved in maintaining redox balance. Binding Nrf2 to ARE induces the expression of these anti-inflammatory genes, primarily NQO1, HO-1 and PRX1 [125,126]. The importance of Nrf2 in the pathogenesis of IBD results from its dual capacity—limiting oxidative stress as described above, but also inhibiting the NF-κB-dependent pro-inflammatory pathway [124,125]. In the course of chronic intestinal inflammation, excessive production of reactive oxygen and nitrogen species (RONS) leads to damage of the intestinal epithelium, disruption of the mucosal barrier integrity, and exacerbation of the immune response [124]. Accumulating evidence indicates that UroA may modulate the activity of the Nrf2 pathway, contributing to the simultaneous reduction in oxidative stress and the inflammatory response [43]. More on the effect of UroA on Nrf2 activity can be found in Section 4.2 and Section 4.3 of this study.

In summary, UroA demonstrates the ability to coordinately modulate key signaling pathways involved in the pathogenesis of IBD, leading to simultaneous inhibition of the pro-inflammatory response mediated by the PI3K/Akt/NF-κB and MAPK/NF-κB pathways, as well as activation of protective mechanisms via the antioxidant and anti-inflammatory Keap1/Nrf2 pathway.

### 5.2. The Effect of Urolithin A on Immune Cell Activity

A growing amount of research indicates that UroA influences the immune response not only by inhibiting the classic pro-inflammatory pathways described above but also by modulating the activity of immune cells, particularly macrophages and T lymphocytes [48,51,54]. Chronic activation of these cells leads to secondary activation of the inflammatory pathway, which in turn results in excessive production of pro-inflammatory cytokines and oxidative stress [110]. UroA is thought to influence immune cells mainly by regulating calcium signaling and post-transcriptional mechanisms associated with microRNAs [48,54].

One of the first studies describing these relationships was the research by Zhang et al., which demonstrated that UroA inhibits the activation and proliferation of CD4+ T lymphocytes by limiting the store-operated calcium entry (SOCE) mechanism [54]. In activated lymphocytes, a decrease in Ca^2+^ concentration in the endoplasmic reticulum is sensed by STIM1 and STIM2 proteins which translocate towards the cell membrane and activate the Orai1 calcium channel, allowing Ca^2+^ ions to enter the cytoplasm. Calcium influx then activates two nuclear transcription factors—NF-κB and NFAT (nuclear factor of activated T cells)—leading to lymphocyte proliferation and the production of pro-inflammatory cytokines [127]. Zhang et al. showed that UroA increases the expression of miR-10a-5p—a microRNA regulating the post-transcriptional expression of STIM1, STIM2 and Orai1—which leads to the inhibition of SOCE and limited CD4+ T cell activation [54].

The importance of calcium signaling was further demonstrated in the 2021 work by Abdelazeem et al., focusing primarily on the activation of bone marrow-derived macrophages (BMDMs), which extends the action of UroA from the adaptive to the innate immune response [48]. The authors demonstrated that UroA tangibly reduces Ca^2+^ ion influx into cells and modulates the expression of microRNAs associated with the inflammatory response—not only the above-described miR-10, but also miR-146a, miR-155 and miR-99b. Thus, their work re-emphasizes that UroA exerts anti-inflammatory effects already at a very early stage of immune cell activation, involving the regulation of gene expression at the post-transcriptional level [48].

Another important aspect of the immunomodulatory effects of UroA is its action on the Treg cell population [51]. In a study by Ghosh et al. conducted in a murine model of DSS-induced colitis, UroA administration increased the number of Treg cells in the intestine and improved their function, which limited the severity of intestinal inflammation. This mechanism seemed to depend on the activation of the AhR/CYP1A1 axis, as in CYP1A1-deficient mice the protective effect of UroA was discernibly attenuated, and AhR-expressing Treg cells showed increased suppressive activity [51]. Because the imbalance between effector lymphocytes and Treg cells is a key element in the pathogenesis of IBD, the ability of UroA to promote a regulatory response may have significant therapeutic implications [51,110].

Taken together, the presented studies indicate that UroA has a multifaceted immunomodulatory effect in IBD by simultaneously limiting the activation of macrophages and T lymphocytes by regulating microRNA expression, inhibiting calcium signaling, and influencing the AhR/CYP1A1 axis, which results in a reduction in pro-inflammatory cytokine production and silencing intestinal inflammation.

### 5.3. The Effect of Urolithin A on Intestinal Barrier Integrity and Tight Junction Proteins

Cytokine-mediated intestinal barrier damage is a key element in the pathogenesis of IBD [110]. The proper functioning of the epithelial barrier depends on the integrity of tight junctions (TJs), formed by proteins such as zonula occludens (mainly ZO-1, ZO-2), occludins and claudins, which regulate the paracellular permeability of the intestinal epithelium [128,129]. In the course of IBD, the organization and expression of TJ proteins are disturbed and the permeability of the intestinal barrier increases, leading to its dysfunction and the development of the so-called “leaky gut”, which promotes commensal bacterial antigens from the intestinal lumen and intensification of the inflammatory response [110,129]. A summary of the molecular landscape of UroA activity in the IBD pathomechanism is presented in Figure 4.

The protective effect of UroA in the treatment of IBD involves restoring proper intestinal barrier function and limiting inflammation induced by tight junction dysfunction. A study by Hering et al. assessed the effect of the ellagitannin punicalagin on intestinal barrier integrity in Caco-2 and HT-29/B6 intestinal epithelial cell models [55]. The authors demonstrated significant differences between the effects of its bioactive metabolites, i.e., EA and UroA. They found that EA enhanced barrier function both in disease and under physiological conditions, whereas UroA primarily demonstrated protective effects in TNF-α-induced inflammation [55]. TNF-α leads to the deregulation of the expression of tight junction proteins, particularly claudin-1 and claudin-2, and to the disruption of their localization within the cell membrane, resulting in increased intestinal barrier permeability [55,130]. Claudin-2 plays a particularly important role in the development of “leaky gut” because it creates channels that increase epithelial permeability to ions and water [130]. A study by Hering et al. showed that UroA limited the TNF-α-induced increase in claudin-1 and claudin-2 expression and prevented claudin-1 delocalization, leading to reduced permeability and the restoration of normal intestinal barrier function [55]. These findings highlight that EA and UroA exert partially distinct biological effects. Therefore, results obtained with punicalagin or EA should not be interpreted as direct evidence of UroA activity, although they provide important insight into the metabolic pathway leading to UroA formation.

The mechanisms responsible for the protective effect of UroA also include activation of the aryl hydrocarbon receptor (AhR), important for maintaining intestinal homeostasis and regulating the mucosal immune response [50,52]. A study by Ma et al. conducted on models of DSS-induced colitis demonstrated that UroA modulates microbial tryptophan metabolism, increasing the production of indole-3-aldehyde (IAld), which in turn activates AhR and enhances IL-22 production [52]. IL-22 plays a crucial role in intestinal epithelial regeneration and maintaining the integrity of the mucosal barrier through the production of mucin 1 and the antimicrobial lectin RegIIIb as well as epithelial cell proliferation [52,110]. In addition to the AhR/IL-22 axis, a second AhR-dependent mechanism, related to CYP1A1 activation, was also described. Ghosh et al. showed that the protective effect of UroA on the intestinal barrier was significantly impaired in CYP1A1-deficient mice, indicating a key role of the AhR/CYP1A1 axis in the action of this compound [52]. The authors suggest that this mechanism may be partially independent of IL-22 and related to the regulation of intestinal epithelial homeostasis and local AhR signaling activity [52].

Furthermore, UroA enhances intestinal barrier integrity by activating the AhR/Nrf2 pathway, leading to increased expression of tight junction proteins such as claudin-4, occludin, and ZO-1 [43]. In a study by Singh et al., the protective effect of UroA on the intestinal barrier was notably attenuated in Nrf2-deficient mice, confirming the crucial role of the AhR/Nrf2 axis in maintaining intestinal epithelial permeability and limiting inflammation. The researchers emphasize that this effect may also result from UroA’s significant role in limiting oxidative damage to TJ proteins [43]. More on the antioxidant effects of UroA through Nrf2 activation can be found in Section 4.2 of this study.

Collectively, the available data indicate that UroA exerts multifaceted protective effects on the intestinal barrier. This compound influences the proper structure, function and composition of TJ proteins by inhibiting TNF-α-related pathways and activating AhR-related pathways, which consequently reduces epithelial permeability and prevents the development of “leaky gut”.

### 5.4. Anti-Inflammatory and Antioxidant Effects of Urolithin A

One of the most important mechanisms of the cytoprotective effect of UroA is activation of the Nrf2 pathway (mentioned earlier in Section 4.1), responsible for regulating the antioxidant response. A study by Singh et al. demonstrated that both UroA and its analogue UAS03 activate the Nrf2 pathway, leading to increased expression of genes encoding antioxidant and detoxification enzymes, including heme oxygenase-1 (HO-1) and NAD(P)H quinone dehydrogenase 1 (NQO1) [43]. Activation of Nrf2 led to a reduction in the production of reactive oxygen and nitrogen species (RONS) and reduced oxidative stress-induced intestinal epithelial cell damage [43]. It has also been shown that activation of Nrf2 by UroA/UAS03 can indirectly inhibit NF-κB signaling, leading to a reduction in the expression of pro-inflammatory mediators such as TNF-α, IL-1β, iNOS, and COX-2 [43,110]. Consequently, the action of UroA includes both the reduction in oxidative stress and the attenuation of the inflammatory response. Because UAS03 is a synthetic analogue of UroA, these findings should be interpreted as supporting the role of Nrf2 activation within this chemical class rather than constituting direct evidence of UroA activity.

Another potential mechanism of UroA’s anti-inflammatory effects is direct modulation of cyclooxygenase-2 (COX-2) activity. In silico and in vitro studies have shown that UroA binds to COX-2 active sites (TYR355, PHE518, ILE517 and GLN192), demonstrating stable affinity for the enzyme and anti-inflammatory activity associated with the inhibition of prostaglandin G2 (PGG2)-dependent processes [53]. In addition to its direct effect on COX-2, UroA may also modulate other pathways related to eicosanoid metabolism. Giménez-Bastida et al., analyzing various types of urolithins, showed that various UroA and iso-UroA reduced the formation of hemiketal eicosanoids HKE2 and HKD2, which are products of the 5-LOX/COX-2 cross-linked enzymatic pathway, in a concentration-dependent manner, whereas urolithin C reduced 5-HETE and LTB4 levels by only inhibiting 5-LOX [56]. Although iso-UroA exhibited comparable activity in this model, these observations should not be considered interchangeable with those obtained using purified UroA. This mechanism may be important in limiting chronic inflammation, as lipid mediators synthesized with 5-LOX and COX-2 participate in the angiogenesis process, including by promoting intestinal microvascular endothelial cell migration, which is a key element in initiating and maintaining inflammation in IBD [59].

Collectively, the available data indicate that UroA has multifaceted anti-inflammatory and antioxidant effects, including both activation of the cytoprotective Nrf2 pathway and modulation of enzymes and mediators involved in maintaining chronic inflammation.

### 5.5. The Induction of Mitophagy and Regulation of the PINK1/Parkin Axis as a Mechanism of the Anti-Inflammatory Action of Urolithin A

Besides the Keap1/Nrf2 pathway described above, the process of mitophagy is responsible for protecting enterocytes from oxidative stress; consequently, mitophagy disorders as well as mitochondrial dysfunction play a significant role in the pathogenesis of IBD [131]. Damaged mitochondria are a source of excessive production of mitochondrial reactive oxygen species (mtROS), which enhance the activation of pro-inflammatory pathways, including NF-κB and the NLRP3/caspase-1 inflammasome, leading to intestinal epithelial damage and the maintenance of chronic inflammation [132,133]. Notably, available data suggest that activation of the Nrf2 pathway and the induction of mitophagy are not completely independent processes but rather mutually reinforcing elements of the cytoprotective response. Activation of Nrf2 promotes the maintenance of redox homeostasis and mitochondrial function, while mitophagy limits the accumulation of damaged mitochondria and mtROS, reducing activation of pro-inflammatory pathways [131].

Under physiological conditions, mitochondrial serine/threonine kinase PINK1 (PTEN-induced kinase 1) is transported into properly functioning mitochondria and undergoes rapid degradation [134]. A loss of mitochondrial transmembrane potential, characteristic of damaged mitochondria, inhibits PINK1 degradation and promotes its accumulation on the outer mitochondrial membrane [57,134]. Accumulated PINK1 phosphorylates ubiquitin and recruits the cytosolic ubiquitin (Ub)–protein ligase Parkin from the cytoplasm to the mitochondrial surface [134,135]. Activated Parkin catalyzes the ubiquitination of numerous outer mitochondrial membrane proteins, leading to the marking of the damaged organelle for autophagic degradation [57,134].

UroA enhances this process by supporting mitochondrial homeostasis and limiting the accumulation of damaged mitochondria that produce mtROS [57]. Although the NIX-dependent pathway was initially considered to be involved in the regulation of mitophagy in IBD (because increased expression of this protein was demonstrated in the inflamed intestinal epithelium), NIX expression was found to be variable and did not clearly correlate with a protective effect of mitophagy [49]. Much stronger evidence, however, was obtained for the Parkin-dependent pathway. Increased expression of its activated form—pSer65-PARKIN—was observed in inflammatory tissues of humans and mice; further, Parkin−/− mice developed more severe DSS-induced colitis, indicating that Parkin-dependent mitophagy plays an important protective role in IBD [49]. Moreover, 7-day administration of UroA in mice increased the expression of both pSer65-PARKIN and NIX; it also improved mitochondrial respiration and reduced intestinal inflammation, suggesting that UroA enhances the adaptive mitophagic response under conditions of chronic inflammation [49].

Translational studies also support the importance of this mechanism. A randomized, placebo-controlled clinical trial demonstrated that UroA supplementation improved mitochondrial function and led to metabolic remodeling of immune cells, including increased mitochondrial biogenesis and the efficiency of CD8+ T lymphocytes by reducing their dependence on glucose [136]. These results suggest that mitophagy activation by UroA may be important not only in aging but also in diseases associated with chronic inflammation, including IBD.

Collectively, the available data indicate that the regulation of mitochondrial homeostasis and the induction of mitophagy are key elements of the multifaceted anti-inflammatory action of UroA. By limiting the accumulation of damaged mitochondria and reducing mtROS, UroA may attenuate the activation of pro-inflammatory pathways and support the maintenance of intestinal barrier integrity.

### 5.6. From Gene Expression to Mitochondrial Homeostasis—A Summary of the Multifaceted Action of Urolithin A in Inflammatory Bowel Diseases

In summary, the available experimental and preclinical data indicate that UroA exhibits exceptionally multifaceted anti-inflammatory effects in IBD, involving virtually all stages of the inflammatory response—from regulating molecular processes and gene expression to modulating effector cell function and the metabolism of inflammatory mediators.

UroA influences the activity of key transcription factors and signaling pathways associated with IBD, such as NF-κB, PI3K/Akt/mTOR, MAPK, AhR and Nrf2, leading to both inhibition of pro-inflammatory gene expression and activation of cytoprotective and antioxidant mechanisms [43,48,50,51,52]. These changes result in reduced production of pro-inflammatory cytokines, reactive oxygen and nitrogen species (RONS), and mitochondrial ROS (mtROS), responsible for maintaining chronic inflammation and intestinal epithelial damage [43,57,109,110]. UroA’s action also encompasses post-transcriptional and post-translational processes, including the regulation of microRNA, the phosphorylation of signaling proteins and activation of the PINK1/Parkin axis, which is associated with mitophagy and the maintenance of mitochondrial homeostasis [48,49,54,120]. This compound simultaneously modulates the activity of immune cells, limiting the activation of macrophages and effector T cells while supporting the Treg-dependent regulatory response [48,52,54]. UroA also influences intestinal barrier function by regulating the expression of tight junction proteins and reducing the “leaky gut” phenomenon [51,55,110,119]. Furthermore, it influences the activity of enzymes involved in eicosanoid metabolism, such as COX-2 and 5-LOX [48,53,59].

This broad spectrum of action means that UroA does not target a single inflammatory mediator but rather modulates an entire complex process of inflammatory response at multiple levels of cellular organization. Combined with the growing number of reports regarding the beneficial effects of UroA in neurodegenerative diseases [137], metabolic disorders [66,138] and liver [93] and kidney [138,139] diseases, this indicates that UroA may represent a promising direction for the development of new therapies supporting the treatment of IBD. The cited research and its details are presented in the table below (Table 1).

**Table 1:** Research on influence of UroA on IBD.

Ref	Experimental Model	UroA Dosage	Duration	Administration Route	Outcomes	Affected Pathways	Limitations
[54]	Naive CD4+ T cells isolated from C57BL/6 murine model	5–50 μM (in vitro)	NS	in vitro culture	↓ T-cell activation and proliferation; alleviated colitis	miR-10a-5p, STIM1/2, Orai1, SOCE	Mainly preclinical; no human validation
[43]	DSS-induced, murine colitis model	NS	NS	Oral	↑ barrier integrity and ↓ inflammation	AhR, Nrf2, HO-1, NQO1, TJ proteins	Animal model only
[50]	DSS-induced, murine colitis model	NS	NS	Oral	attenuated colitis severity and restored tryptophan metabolism	AhR signaling, tryptophan metabolism	No human validation
[51]	DSS-induced, murine colitis model	NS	NS	Oral	↓ intestinal inflammation and ↑ immune homeostasis	AhR activation, Treg regulation, cytokine modulation	Preclinical study
[48]	BMDMs	25–50 μM	24 and 48 h	In vitro	↓ ROS production and inflammatory mediators	PI3K/Akt, MAPK, NF-κB, calcium signaling	Macrophage model only
[49]	DSS-induced, murine colitis model	NS	7 days	Oral	↓ colitis severity, improved epithelial barrier function, ↓ intestinal inflammation	AhR signaling, epithelial regeneration, mucosal homeostasis	Preclinical study; no human validation
[136]	Human clinical trial	1000 mg/day	28 days	Oral	↑ immune cell mitochondrial function; ↓ inflammatory markers	Mitophagy, mitochondrial metabolism	Not performed in IBD patients

Abbreviations: AhR—aryl hydrocarbon receptor, BMDMs—bone marrow-derived macrophages, CD4+—cluster of differentiation 4, DSS—dextran sulfate sodium, HO-1—heme oxygenase-1, IBD—inflammatory bowel disease, MAPK—mitogen-activated protein kinase, NF-κB—nuclear factor kappa-light-chain-enhancer of activated B cells, NQO1—NAD(P)H quinone dehydrogenase 1, Nrf2—nuclear factor erythroid 2-related factor 2, NS – not specified, PI3K/Akt—phosphoinositide 3-kinase/protein kinase B, ROS—reactive oxygen species, SOCE—store-operated calcium entry, STIM1/2—stromal interaction molecule ½, TJs—tight junctions, Treg—regulatory T cell, and UroA—urolithin A; ↑—indicates increase/improve; ↓—indicates decrease/reduce.

## 6. Anti-Neoplasm Properties of Urolithin A

### 6.1. Mechanisms of Carcinogenesis in Colorectal Cancer

Colorectal cancer (CRC) poses a significant global health challenge, as current epidemiological data indicate a steady increase in its incidence rates in the coming decades [140]. The multifactorial etiology of CRC allows for the classification of risk factors into two primary categories, where modifiable factors include a diet high in processed foods, tobacco smoking, excessive alcohol consumption, obesity, and a sedentary lifestyle [141]. In contrast, non-modifiable factors involve genetic predispositions and hereditary syndromes, such as familial adenomatous polyposis [142]. The progression of CRC typically takes several years and most frequently originates from precancerous adenomatous polyps, which provides a time window for potential prophylactic interventions. Clinical manifestations frequently include persistent abdominal pain, unexplained weight loss, alterations in bowel habits, the presence of blood in stool, or iron deficiency anemia [143].

Current CRC therapeutic strategies are mostly based on surgical resection, systemic chemotherapy, and immunotherapy [144]. Modern oncology focuses on the development of personalized, targeted therapies designed to maximize cytotoxic or cytostatic efficacy while minimizing systemic toxicity and reducing the risk of disease recurrence [145]. Because advanced malignant cells exhibit a capacity to evade classical apoptotic triggers and cell cycle checkpoints, new chemotherapeutic agents are expected to affect the cells at the molecular level. In this context, UroA has emerged as a potent antineoplastic agent capable of counteracting the processes occurring in human CRC models [59]. The comprehensive network of these targeted intracellular interactions and antitumorigenic mechanisms is schematically summarized in Figure 5.

### 6.2. UroA on Autophagy in Colorectal Cancer

One of the mentioned mechanisms crucial in CRC pathogenesis is autophagy. It is a critical cellular degradation and recycling process that exerts a paradoxical, stage-dependent influence on CRC progression [119,146]. During the initial stages of oncogenesis, autophagy functions as a tumor suppressor by clearing damaged intracellular organelles, preventing the accumulation of reactive oxygen species, and maintaining genomic stability. This role undergoes a fundamental shift toward tumor promotion as cells become more malignant. The CRC tumor microenvironment is frequently subjected to severe hypoxia and nutrient deprivation. Autophagy alleviates this metabolic stress by recycling intracellular components, which sustains rapid cellular proliferation. This response also conditions significant chemoresistance. As a result, standard cytotoxic agents used in CRC management can paradoxically enhance tumor survival by hyperactivating protective autophagic pathways [146].

The molecular orchestration of autophagy in CRC is based on an integrated signaling cascade rather than isolated pathways [146]. The PI3K/Akt signaling axis acts as a primary negative regulator by stimulating mTORC1. Active mTORC1 suppresses autophagy initiation via the direct phosphorylation and catalytic inhibition of the ULK1 complex. As cellular energy levels decline, robust AMPK activation occurs. This protein facilitates autophagosome formation through two distinct mechanisms: the direct functional suppression of mTORC1 and the activating phosphorylation of ULK1. Collectively, these signaling events initiate autophagy. Following these initiation events, the assembly of the Beclin-1 and class III PI3K complex is strictly necessary for the nucleation of the phagophore and subsequent autophagosome maturation. To elongate the newly formed membrane, conjugation systems must lipidate LC3-I protein into membrane-bound LC3-II, providing the necessary scaffold for autophagosome completion.

One of the anti-neoplastic UroA features lies in the ability to hyperactivate autophagy. Evidence demonstrates that this compound functions as an autophagic inducer in CRC cells even at submicromolar concentrations [58]. In the SW620 cell line model, UroA treatment triggers substantial expression and accumulation of LC3, which leads to the assembly of autophagosomes. This autophagic induction is tightly linked to the targeted suppression of the AKT and WNK1 signaling axis. UroA has been shown to inhibit the phosphorylation of both kinases [60]. Because the active AKT/WNK1 axis inversely correlates with intracellular LC3 levels, its pharmacological suppression by UroA directly stimulates LC3 production and accelerates autophagic flux [16]. Considering the simultaneous apoptotic engagement [58], this response may synergize with the apoptotic signaling role in UroA tumor-suppressive effects across progressive CRC stages.

### 6.3. UroA on Cellular Senescence in Colorectal Cancer

Cellular senescence exerts a double-phased, stage-dependent influence on CRC progression—similarly to autophagy. During early oncogenesis, it functions as a potent tumor-suppressive mechanism by inducing stable cell cycle arrest in pre-malignant cells, primarily mediated by the p53 and p16 pathways [147]. In contrast, chronic accumulation of senescent cells within the tumor microenvironment drives tumor promotion. This shift is mediated by SASP, which conditions localized inflammation, facilitates metastasis, and induces therapeutic resistance [148]. Current pro-senescence strategies show promising clinical potential. Importantly, it is necessary to use senolytic agents to clear senescent cells and reduce SASP sequentially, as it helps prevent unwanted tumor growth [149].

UroA drives malignant cells into a state of stable senescence, phenotypically confirmed by a marked increase in senescence-associated beta-galactosidase (SA-beta-gal) activity [61]. This influence can be explained by direct activation of the p53-dependent signaling cascade. In wild-type p53 models, such as the HCT-116 cell line, UroA effectively inhibits cellular proliferation and enforces an irreversible senescent state [56]. This pharmacological response is dependent upon p53 functionality, as CRC lines harboring p53 mutations, as well as nontumorigenic control cells, do not exhibit a comparable senescent shift. These findings suggest a degree of selectivity of UroA toward p53-competent CRC cells. UroA can also be distinguished from conventional chemotherapeutic agents on the basis of selective engagement with the senescence pathway. Such interaction offers a targeted approach to permanently arrest tumor growth, instead of relying solely on the induction of acute apoptosis—which advanced tumors frequently resist [150,151].

### 6.4. UroA on Apoptosis in Colorectal Cancer

Paralleling the tumor-suppressive functions of cellular senescence, apoptosis constitutes another critical biological barrier to CRC initiation that malignant cells systematically evade. This tightly regulated process relies on the execution of intrinsic and extrinsic pathways that converge upon caspase cascade activation [63]. The intrinsic signaling is triggered by intracellular stress factors such as DNA damage or severe oxidative stress. This results in Bax-mediated mitochondrial outer membrane permeabilization and subsequent cytochrome c efflux. The resulting apoptosome complex with Apaf-1 facilitates caspase-9 activation, directly cascading to the executioner caspase-3. The extrinsic pathway operates via death receptors like Fas, which initiate caspase-8 activation. Protein tBid connects these pathways by amplifying mitochondrial permeabilization, leading to final cell dismantling through the cleavage of substrates like PARP [62].

By engaging apoptotic pathways, UroA directly compromises CRC viability, as it functions as a potent apoptosis inducer through diverse molecular mechanisms. For instance, it has been noted that the expression of the initiator caspase-9 and the effector caspase-3 can be upregulated by UroA, resulting in apoptosis facilitation [62,63]. Experimental models demonstrate that UroA treatment induces PARP cleavage, which serves as a biochemical marker of apoptotic execution [63]. At the same time, UroA suppresses anti-apoptotic proteins including Bcl-2 while inducing pro-apoptotic factors such as p53, p21, and Bax [59,63]. These molecular alterations shift the intracellular balance toward programmed cell death, resulting in CRC tumor growth inhibition. This apoptotic response is primarily mediated via a p53-dependent axis, particularly evident in CRC cell lines with wild-type p53 where elevated p21 expression reinforces cell cycle arrest and subsequent apoptosis [59]. Importantly, these pro-apoptotic properties act synergistically with other antineoplastic mechanisms of UroA like autophagy [58] and ROS generation [62,63].

### 6.5. UroA on Cell Cycle in Colorectal Cancer

Beyond the evasion of apoptosis, malignant transformation requires the subversion of cell cycle checkpoints to sustain unrestricted cellular proliferation. Inactivating mutations in key regulatory proteins disrupt normal control systems, leading to unchecked division. Progression through the cycle depends entirely on the balance between CDKs, cyclins, and their inhibitors [152,153].

When evaluating the anticancer efficacy of UroA against CRC, several specific modulators are important. The CDK6-cyclin D complex drives the G1/S transition and initiates DNA replication [152]. Subsequent S/G2 progression relies on cyclin A to ensure accurate genomic duplication and reduce replication stress. The G2/M transition is regulated by cyclin B, and its dysregulation causes mitotic instability, accelerating oncogenesis [153]. Conversely, the p21 protein is a potent CDK inhibitor. It halts the cell cycle during cellular stress by blocking kinase activity across transitions like G1/S and G2/M [153].

Complementing its pro-apoptotic effects, UroA disrupts malignant proliferation through comprehensive cell cycle modulation. It acts as a broad-spectrum regulator that targets multiple interphase transitions. By significantly upregulating p21 expression, UroA directly inhibits CDK activity and effectively arrests cycle progression at the G1/S checkpoint [62]. This specific inhibitory response functions predominantly in CRC cell lines harboring wild-type p53 [59]. Furthermore, UroA blocks the G2/M transition by driving the downregulation of cyclin B1 and CDK1, both of which are strictly required for mitotic entry [46]. The sustained elevation of p21 levels further suppresses the catalytic function of CDK1 to enforce a strict G2/M blockade.

### 6.6. UroA on Metastatic Dissemination in Colorectal Cancer

Unrestricted cell proliferation driven by cell cycle dysregulation forces primary tumor expansion and sets the stage for metastatic dissemination. In advanced clinical stages, CRC frequently metastasizes to distant sites, predominantly the liver and peritoneum [154]. This process is driven by extensive cellular reprogramming that depends heavily on EMT and the proteolytic activity of MMPs.

EMT alters the cellular phenotype by reducing the apical–basal polarity of the cells and intercellular adhesion between them while promoting mesenchymal traits that enhance motility and invasiveness [155]. This phenotypic shift involves the modulation of specific cellular markers. Consequently, EMT not only drives spatial dissemination but also aggravates systemic chemotherapy resistance [156]. The molecular regulation of this process relies on canonical signaling pathways, including TGF-beta, NF-kappaB, and Wnt/beta-catenin, which converge on transcription factors like Snail and Slug [157].

Simultaneously, the physical degradation of ECM facilitates active tissue invasion. This proteolytic remodeling is governed by MMPs, with MMP-2, MMP-7, and MMP-9 being the most crucial [158]. These endopeptidases further amplify tumor cell migration by interacting directly with localized growth factors and cell adhesion networks [159].

UroA impairs this metastatic cascade by targeting the plasticity of CRC cells. Studies indicate that UroA modulates extracellular matrix remodeling, thereby decreasing the invasive capacity of the tumor. Research involving diverse CRC cell lines demonstrates that UroA treatment significantly attenuates the expression and proteolytic activity of MMP-1, MMP-2, and MMP-9 [160]. These observations are supported by independent in vitro investigations [58]. Alongside suppressing MMPs, UroA upregulates endogenous tissue inhibitors of metalloproteinases (TIMPs), particularly TIMP-1 [160]. This favorable shift in the MMP-to-TIMP ratio neutralizes localized matrix degradation, tightly restricting the physical invasion of malignant cells.

Parallel to its anti-proteolytic functions, UroA inhibits the epithelial-to-mesenchymal transition (EMT). Notably, it suppresses its signaling even in 5-fluorouracil (5-FU)-resistant CRC lineages, promoting a reversion to a highly structured, less invasive epithelial phenotype [51]. At the molecular level, UroA upregulates key junctional proteins, including E-cadherin and ZO-1, while downregulating mesenchymal transcription factors such as Snail and beta-catenin. This molecular shift successfully limits the migratory capabilities of the tumor. Ultimately, this targeted suppression of cellular plasticity is a vital mechanism for reversing acquired chemoresistance and resensitizing refractory CRC to standard cytotoxic regimens, a broad therapeutic benefit similarly confirmed across diverse oncological models [139].

### 6.7. Summary of UroA Influence on CRC Progression

In summary, CRC progresses by evading normal cellular defenses, such as apoptosis, cell cycle checkpoints, and structural barriers. Available evidence suggests that UroA may counteract several mechanisms involved in CRC progression. It limits primary tumor growth and metastasis by inducing senescence and arresting the cell cycle. Simultaneously, UroA reduces cell survival through p53-dependent apoptosis and excessive autophagy. By inhibiting the epithelial-to-mesenchymal transition and protecting the extracellular matrix from degradation, UroA disrupts tumor stability. Ultimately, these combined actions neutralize CRC survival strategies and show great potential for resensitizing resistant tumors to therapy. The cited research and its details are presented in the table below (Table 2).

**Table 2:** Research on influence of UroA on CRC.

Ref	Experimental Model	UroA Dosage	Duration	Administration Route	Outcomes	Affected Pathways	Limitations
[60]	HuCCT-1 and SSP-25 cell lines	test-dependent: 10 or 40 μmol/L	test-dependent: 3 h up to 72 h	mixed to medium	induces autophagy	inhibition of phosphorylation of AKT and WNK1 kinases	- low oral bioavailability - lower bioactivity of phase II metabolites
	Female nude mice (BALB/c Slc-nu/nu)	20 mg/kg, 3 times a week	35 days	p.o.			
[139]	SW480, HCT-116 and 5FUR cell lines	test-dependent: 0, 5, 10, 15, 20 or 25 μmol/L	test-dependent: 5 h up to 24 h	mixed to medium	resensitizes refractory tumors to standard cytotoxic regimens	induction of degradation of Snail protein by stabilizing p53; subsequent ↑ mdm2 to mediate Snail ubiquitination	- no glucuronidation interaction studies
[51]	5-FU-resistant and 5-FU-susceptible HCT-116 and SW-480 cell lines	test-dependent: 10 or 50 μmol/L	test-dependent: 24, 48 or 72 h	mixed to medium	chemoresistance reversal; EMT inhibition (with 5-FU); reduced migration; epithelial phenotype promotion; apoptosis induction	↑ E-cadherin and ZO-1; ↓ Snail and beta-catenin; regulation of FOXO3-FOXM1 axis	- no glucuronidation interaction studies - ineffective as monotherapy in vivo
	NRGS and C57BL/6 wild type (WT)	20 mg/kg test-dependent: 2 or 3 times a week	test-dependent: 6 weeks and 3 weeks	UroA: p.o. 5-FU: i.p.			
[58]	SW-620 cell line	test-dependent: 0.15, 1.5, 15 or 30 μmol/L	test-dependent: 24, 48 or 72 h	mixed to medium	induces autophagy and apoptosis; inhibits metastasis	suppression of AKT/WNK1 signaling axis; LC3 expression and accumulation, MMP-2 and MMP-9 suppression	- no in vivo validation - no glucuronidation interaction studies - dual role of autophagy
[63]	SW-480, HT-29 and SW-620 cell lines	test-dependent: 0, 3.125, 6.25, 12.5, 25, 50, 100, or 200 μmol/L	test-dependent: 24 or 48 h	mixed to medium	induces cell cycle arrest and apoptosis; induces PARP cleavage	↑ caspase-9, caspase-3, p53, p21, and Bax; suppression of Bcl-2; ROS generation	not specified
[56]	HCT-116 cell line (wild-type p53), p53-mutated CRC lines, and nontumorigenic control cells	test-dependent: 0.5, 1 or 10 μmol/L	test-dependent: up to 5 or 14 days	mixed to medium	inhibits cellular proliferation; enforces irreversible senescent state	direct activation of p53-dependent signaling cascade; increase in SA-beta-gal activity	- p53-dependent senescence - lower bioactivity of phase II metabolites - risk of tumor recurrence due to reversible senscence
[160]	HT-29, SW480 oraz SW620	test-dependent: 25, 50 or 100 μmol/L	test-dependent: 24 or 48 h	mixed to medium	decreases invasive capacity; modulates extracellular matrix remodeling	attenuates expression and proteolytic activity of MMP-1, MMP-2, and MMP-9; ↑ TIMP-1	- no in vivo validation - no glucuronidation interaction studies

Abbreviations: 5-FU—5-fluorouracil, 5FUR—5-fluorouracil-resistant cell line, AKT—protein kinase B (Akt), Bax—Bcl-2-associated X protein, Bcl-2—Bcl-2 apoptosis regulator protein, CRC—colorectal cancer, E-cadherin—epithelial cadherin, EMT—epithelial–mesenchymal transition, FOXM1—Forkhead box M1, FOXO3—Forkhead box O3, h—hours, i.p.—intraperitoneally, LC3—microtubule-associated protein 1 light chain 3, mdm2—mouse double-minute 2 homolog, mg/kg—milligram(s) per kilogram of body weight, MMP-1—matrix metalloproteinase-1, MMP-2—matrix metalloproteinase-2, MMP-9—matrix metalloproteinase-9, NRGS—NOD-Rag1null IL2rgnull mice expressing human cytokines (immunodeficient mouse strain), p.o.—per os (orally), p21—protein p21 (cyclin-dependent kinase inhibitor 1), p53—tumor protein p53, PARP—poly (ADP-ribose) polymerase, ROS—reactive oxygen species, s.a.—such as, SA-beta-gal—senescence-associated beta-galactosidase, TIMP-1—tissue inhibitor of metalloproteinases, UroA—urolithin A, WNK1—WNK lysine-deficient protein kinase 1, WT—wild type, ZO-1—zonula occludens-1, and μmol/L—micromoles per liter; ↑—indicates increase; ↓—indicates decrease.

## 7. Limitations of Urolithin A-Based Therapy and Future Research Directions

Despite promising preclinical results and the fact that the spectrum of action of UroA is the best characterized of all known urolithins, its use in IBD and CRC therapy still presents numerous limitations [161]. However, it should be noted at the outset that available clinical studies indicate that oral UroA supplementation is generally safe and well tolerated. Two multicenter clinical trials were conducted in 2022, the results of which showed that after four months of UroA supplementation, no serious safety concerns were reported at doses up to 1000 mg/day [162,163]. One of the most important problems remains the limited bioavailability of ellagitannins, from which UroA is produced in the body. This is due to the low solubility of these compounds in gastrointestinal fluids, which makes it difficult to achieve sufficient concentrations at target sites after oral intake [161]. Another pharmacokinetic problem is the high interindividual variability in endogenous UroA production from ellagitannins, which depends on the composition of the gut microbiota [117,161]. UroA is not supplied directly in the diet, but it is a product of ellagitannin metabolism by specific intestinal bacteria, the presence and activity of which vary between patients [117]. Three main urolithin metabotypes have been described (UM-A, UM-B, and UM-0), differing in their ability to produce UroA; however, some people (UM-0) do not synthesize urolithins at all [161]. In the context of IBD, associated with profound dysbiosis, this may significantly limit the effectiveness of therapies based only on dietary precursors rich in ellagitannin, i.e., berries, grapefruits, grapes, seeds and nuts [109,117]. Therefore, it seems sensible to directly supplement UroA, probiotics capable of producing UroA or its more stable analogues (more on these below), which would overcome both the problem of intestinal dysbiosis and the limited bioavailability of precursors [118,161]. Additionally, the dominant circulating form of UroA is phase II metabolites (glucuronides and sulfates), the biological activity of which may be limited relative to the free UroA form [43,56]. Giménez-Bastida et al. showed that conjugated metabolites of urolithins, including UroA, were practically inactive in terms of modulating COX-2 and 5-LOX-dependent eicosanoid pathways, which may limit the therapeutic anti-inflammatory efficacy of UroA in vivo [59]. Therefore, an important issue that requires consideration is the interpretation of these study results in light of the concentrations used in experimental models. Many studies of UroA-mediated inhibition of CRC cell proliferation or induction of apoptosis have used concentrations ranging from 10 to 50 μM, which may exceed plasma concentrations achieved after ingestion [58]. However, because UroA is produced and accumulates locally in the intestinal lumen (as mentioned above), its concentrations in colonic tissues may be significantly higher than systemic concentrations, potentially increasing the translational significance of these results [117,161]. Further pharmacokinetic and tissue distribution studies are necessary to determine whether the concentrations effective in experimental CRC models can be achieved in humans.

Another notable limitation is that most available data come from experimental models of DSS-induced colitis or in vitro studies, while large clinical trials assessing the efficacy and safety of long-term UroA use in patients with IBD and CRC are still lacking. It is also not fully understood which mechanisms of UroA action have dominant therapeutic importance in the conditions of human chronic intestinal inflammation, especially in the context of complex interactions between the microbiota, the immune system and mitochondrial metabolism [110]. Moreover, another problem is the previously mentioned fact that UroA and its phase II metabolites, as the main circulating forms, are the most thoroughly studied members of the urolithin family, while currently much less is known about the biological activity of other microbiome-derived metabolites, including UroB and iso-UroA [43,59,161]. Future studies directly comparing the biological effects of individual urolithins and their circulating conjugated forms may provide a more comprehensive understanding of their therapeutic potential. At the same time, recent research has significantly advanced potential strategies for increasing the effectiveness of UroA-based therapies. The use of nanotechnology and synthetic analogues of UroA appears to be a particularly interesting direction [43,118]. Ghosh et al., in studies on murine models of DSS-induced colitis, developed nanoparticles containing UroA and its synthetic analogue UAS03, which both selectively accumulated in inflamed intestinal tissue and reduced the severity of colitis more effectively than free UroA. Importantly, the therapeutic effect was achieved at lower concentrations and less frequent dosing of the compound, which may be a solution to improve bioavailability and reduce potential side effects of UroA [118]. Additionally, Singh et al. demonstrated that the UAS03 analogue retains the biological activity of UroA while exhibiting greater stability and potency. This suggests that UroA may not only be a potential natural drug but also a starting point for the design of new molecules with more beneficial pharmacokinetic properties and greater anti-inflammatory efficacy [43]. Furthermore, modern delivery systems such as pH-driven liposomal encapsulation are being considered, which may increase the compound’s stability, limiting its rapid phase II metabolism, and enable more selective distribution to inflamed tissues. However, due to the poor number of in vivo studies, the effectiveness of this strategy requires further validation before potential implementation in therapeutic practice [161,164].

In the future, it will be crucial to conduct translational and clinical studies to increase the bioavailability and determine the optimal dosage and safety of long-term use of UroA in patients with IBD. It is also crucial to validate molecular pathways responsive to UroA in human tissues from IBD and CRC patients and to conduct additional studies using intestinal organoids, epithelial barrier models, and physiologically relevant concentrations of UroA that may improve translational relevance. Moreover, combining UroA-based therapies with gut microbiota modulation and modern targeted drug delivery systems also seems particularly interesting for further research [164].

## 8. Conclusions

Urolithins are products of the biotransformation of ellagitannins by the gut microbiota which appear to be promising postbiotics. UroA, the best-known member of this group of compounds, shows significant potential in the prevention and adjuvant treatment of chronic IBD and CRC. Current data indicate that the broad biological activity of UroA encompasses not only antioxidant effects but also the modulation of signaling pathways associated with the inflammatory process, regulation of the immune response, improvement in intestinal barrier integrity, and restoration of mitochondrial homeostasis through the induction of mitophagy. The multifaceted mechanisms of action of UroA may contribute to alleviating chronic inflammation and limiting neoplastic processes within the gastrointestinal tract.

Importantly, the biological activity of UroA is closely linked to the composition and metabolic activity of the gut microbiota. Experimental studies have also shown that this compound can inhibit cancer cell proliferation, induce apoptosis, and modulate the molecular pathways involved in the development and progression of CRC, suggesting its potential use as an adjunctive therapeutic strategy.

Despite promising results from preclinical studies, there are still many limitations associated with the potential use of UroA, such as interindividual variability in urolithin production, individually varied gut microbiota composition, a limited number of clinical trials, and insufficient knowledge regarding the long-term effects of a diet rich in ellagitannins. Therefore, further well-designed clinical trials are necessary to evaluate the therapeutic efficacy, bioavailability, and potential for personalized use of UroA in IBD and CRC. Importantly, although substantial mechanistic evidence supports the biological activity of purified UroA, some available studies employ ellagitannins, ellagic acid, pomegranate extracts, or structurally related urolithins. These findings provide valuable biological context but should not be interpreted as direct evidence of UroA-specific effects. Deepening our understanding of the interactions between dietary polyphenols, the gut microbiota, and host signaling pathways may contribute to the development of new therapeutic strategies targeting the gut microbiota in chronic inflammatory and neoplastic diseases. 

## Data Availability

No new data were created or analyzed in this study.
